# Complete Genome Sequences of Three Clinical Isolates of Dengue Virus Serotype 1 from South Korean Travelers

**DOI:** 10.1128/genomeA.01388-15

**Published:** 2015-11-25

**Authors:** Yun Young Go, Eunhye Jung, Young Eui Jeong, Udeni B. R. Balasuriya

**Affiliations:** a Virus Research and Testing Group, Division of Drug Discovery Research, Korea Research Institute of Chemical Technology, Yuseong, Daejeon, South Korea; b Division of Arboviruses, Korea National Institute of Health, Osong, South Korea; c Department of Veterinary Science, Maxwell H. Gluck Equine Research Center, College of Agriculture, Food and Environment and Department of Microbiology, Immunology and Molecular Genetics, College of Medicine, University of Kentucky, Lexington, Kentucky, USA

## Abstract

In this study, we report the complete genome sequences of three clinical isolates of dengue virus serotype 1 isolated from South Korean travelers returning from different countries in Southeast Asia. The nucleotide sequence identities ranged from 91.5 to 92.2%, while the amino acid sequence identities ranged from 97.5 to 97.9% among the three clinical isolates.

## GENOME ANNOUNCEMENT

Dengue virus (DENV) is composed of four distinct serotypes (DENV-1 to -4), which belong to the genus *Flavivirus* in the family *Flaviviridae* (1). DENVs cause a wide range of clinical symptoms in humans, from acute febrile illness dengue fever to life-threatening dengue hemorrhagic fever/dengue shock syndrome (2). Dengue fever is considered a major public health problem in developing tropical countries, where the virus is endemic, and it is continuously spreading to new geographical areas around the world (3, 4). With increasing international travel to tropical regions where dengue is endemic or epidemic, the risk of DENV infection to travelers and expansion of its geographic distribution are continuously escalating (5, 6). The three clinical DENV-1 isolates used in this study were isolated from such imported cases (7). Based on the travel history, DenKor-01, DenKor-02, and DenKor-07 were isolated from travelers who returned from India-Singapore, Indonesia, and the Philippines, respectively.

Using a previously described protocol, the viruses were reverse-transcription PCR (RT-PCR) amplified in five overlapping fragments that covered the complete polyprotein (8). Both sense and antisense strands of each fragment were sequenced at a commercial sequencing facility (GenoTech, Daejeon, South Korea). The 5′ and 3′ terminal sequences of each viral genome were determined by rapid amplification of cDNA ends (RACE) using the FirstChoice RLM-RACE kit (Ambion, Austin, TX, USA), according to the manufacturer’s instructions. The sequencing results were assembled using CodonCode Aligner version 4.1.1 (CodonCode, Inc., Centerville, MA, USA) and further analyzed using the Geneious 6.1.8 (Biomatters Ltd., Auckland, New Zealand) software.

The complete genome sequences of the DenKor-01 (imported case in 2004), DenKor-02 (2005), and DenKor-07 (2006) isolates from the sera of travelers returning from Southeast Asia were 10,736, 10,735, and 10,734 nucleotides in length, respectively, and consisted of a single open reading frame (ORF), which encodes a 3,392-amino acid polyprotein. The nucleotide sequence identities for the complete genomes, including the 5' and 3' untranslated regions (UTR), ranged from 91.5 to 92.2%, while the amino acid sequence identities for the translated regions ranged from 97.5 to 97.9% among the three clinical DENV-1 isolates. Surprisingly, DenKor-01 possessed an extra adenosine (nucleotide position 10,302), while DenKor-07 had a nucleotide (adenosine) deletion (nucleotide position 10,464), both located at the 3' UTR; thus, the length of the polyprotein remained equal in all three viruses. Phylogenetic analysis of the complete genome sequence of DenKor-01 confirmed that this virus belongs to genotype V, together with DENV-1 strains from India and Thailand (GenBank accession numbers JQ922545, AY732476, and AY732474). Similarly, the DenKor-02 isolate was grouped with the genotype I viruses and was very closely related to a strain from Singapore (GenBank accession FJ469909). Last, DenKor-07, isolated from a traveler returning from the Philippines, was included in genotype IV and showed a very close relationship to DENV-1 from Hawaii (GenBank accession no. DQ672564).

### Nucleotide sequence accession numbers.

The assembled complete genome sequences of DenKor-01, DenKor-02, and DenKor-07 strains are deposited in GenBank under the accession numbers KP406801, KP406802, and KP406803, respectively.
